# Physicochemical Properties and the Gelation Process of Supramolecular Hydrogels: A Review

**DOI:** 10.3390/gels3010001

**Published:** 2017-01-01

**Authors:** Abdalla H. Karoyo, Lee D. Wilson

**Affiliations:** Department of Chemistry, University of Saskatchewan, 110 Science Place, Saskatoon, SK S7N 5C9, Canada; abk726@mail.usask.ca

**Keywords:** gel, sol, aggregation, cyclodextrin, hydration

## Abstract

Supramolecular polysaccharide-based hydrogels have attracted considerable research interest recently due to their high structural functionality, low toxicity, and potential applications in foods, cosmetics, catalysis, drug delivery, tissue engineering and the environment. Modulation of the stability of hydrogels is of paramount importance, especially in the case of stimuli-responsive systems. This review will update the recent progress related to the rational design of supramolecular hydrogels with the objective of understanding the gelation process and improving their physical gelation properties for tailored applications. Emphasis will be given to supramolecular host–guest systems with reference to conventional gels in describing general aspects of gel formation. A brief account of the structural characterization of various supramolecular hydrogels is also provided in order to gain a better understanding of the design of such materials relevant to the nature of the intermolecular interactions, thermodynamic properties of the gelation process, and the critical concentration values of the precursors and the solvent components. This mini-review contributes to greater knowledge of the rational design of supramolecular hydrogels with tailored applications in diverse fields ranging from the environment to biomedicine.

## 1. Introduction

Polymer gels are generally defined as 3D networks swollen by a large amount of water [1]. In particular, polysaccharide-based hydrogels are important due to their diverse chemical structure and rich functionality [2,3]. In general, gels play a vital role in the biomedical field (e.g., contact lenses and ocular implants, wound dressing, tissue regeneration and engineering) [4,5,6,7,8], in the environment [9,10,11,12], food processing [13] and personal care products such as cosmetics and disposable diapers [14]. Most recently, polysaccharide-based hydrogels have found application in agriculture as controlled-release devices for fertilizers and agrochemicals [15]. Additional applications include the use of self-healing pH sensitive superabsorbent polymers to self-seal cracks in concrete [16,17]. A number of responsive hydrogel systems based on natural and synthetically modified polysaccharides have been reported. For example, significant contributions on responsive systems are reported by the research groups of Rinaudo [18,19,20,21], Saito [22], and others [5,6,23,24,25]. Polysaccharides are abundant and readily available from renewable sources such as plants and algae, and various microbial organisms [26]. Such polysaccharides have a large variety of compositional and structural properties, making them facile to produce and versatile for gel formation as compared with synthetic polymers. Selected examples of natural polysaccharides for the preparation of stimuli-responsive hydrogels are listed in Table 1.

In contrast to conventional gel formation, the combined use of polymer chains along with stable and selective supramolecular cross-links offer versatile constructs that afford facile modification of structural parameters of the polymer backbone that include the strength and dynamics of cross-linking interactions, and responsiveness to multiple stimuli [27]. Supramolecular hydrogels (or aqua gels) are hydrophilic materials which undergo self-assembly to form 3D continuous networks of macromolecules, where water resides within the interstitial domains of the polymer network [28,29]. Supramolecular hydrogels are a relatively new class of soft and responsive materials of great research interest owing to the broad application of such systems in areas that range from tissue engineering and carrier systems [30] to environmental remediation [31]. The formation of supramolecular materials through host–guest interactions is a powerful method to create non-conventional stimuli-responsive hydrogels. This relates to the host–guest interactions present which can be modulated to fine tune the stability and responsiveness of the resulting gel system based on the choice of macromolecular scaffold. Numerous studies have been reported on supramolecular hydrogels that show reversible response to environmental stimuli; however, the responsive behaviour of many of these materials often depend on the inherent properties of the building blocks rather than the resulting supramolecular interactions. Examples of inherent responsive hydrogel systems include the temperature-induced *rod-to-coil* transition of poly(*N*-isopropyl acrylamide) (PNIPAM) [32,33,34] and oligo(ethylene glycol)s (OEGs) [35,36], the pH induced-protonation of poly(vinyl pyridine) [37,38], and the photosensitive behaviour of azobenzenes [4,39,40].

Host–guest carbohydrate-based hydrogels that employ macrocyclic building blocks such as cyclodextrins (CDs) as porogens [48,52,53,54,55,56,57] are unique for various reasons: (i) tunable physicochemical properties (e.g., mechanical stability, viscosity, etc.); (ii) specificity and effectiveness of the host–guest molecular recognition that lead to stable hydrogel structures; and (iii) wide applications of host–guest hydrogels in various fields such as the environment, biomedicine, delivery systems, and food technology. In general, the formation of supramolecular-based hydrogels occur *via* two processes; either through self-assembly of monomer units to form aggregates that gelate in aqueous solvents, or derived from polymer units and/or host–guest interactions *via* multi-component inclusion systems that contain macromolecular scaffolds [19,51]. The latter is used to form hydrogels based on CD macromolecules as the key building block. Unlike chemical gels which involve covalent bonding (e.g., divinyl sulfone cross-linked gellan gels [58]), physical gel formation occurs *via* multiple, weak non-covalent interactions (e.g., H-bonding, π–π and van der Waals interactions, and hydrophobic effects) [51,59].

Despite recent research advances in physical supramolecular hydrogels, the rational design of such systems is sparsely reported. This knowledge gap poses a significant challenge for the use of these materials for specific applications. Specifically, the optimization of the strength of supramolecular hydrogels is important, especially in the case of stimuli-responsive hydrogels. For example, temperature sensitive hydrogels can encapsulate drugs during the gelation process and the duration or rate of their release at the site of action will depend on the stability of the hydrogels [60]. Thus, the tunability and responsive nature of such materials depend on several factors, such as the solute–solute and solute–solvent interactions, for the gelation process. Yui et al. [61,62] and Zhao et al. [63] have reported an approach for improving the stability of hydrogels by tuning the hydrophile–lipophile balance (HLB) of the precursor materials as a way of stabilizing the macromolecular assembly. The stability of the supramolecular assembly can be tuned by varying the nature and relative feed ratios of the host/guest system or the precursor materials [23]. Furthermore, the role of the aqueous solvent on the hydrogel stability is significant [20], according to the HLB of the system. This mini-review presents a coverage of the literature in the past five years concerning the physicochemical properties and structural variables that can be tuned to improve the formation and stability of hydrogels for tailored applications. Examples of spectroscopic (e.g., NMR, XRD, and FT-IR) and microscopic (e.g., TEM and SEM) studies related to the rheological and structural information of the hydrogel assembly will be presented in order to gain a greater understanding of the rational design of these materials. In particular, the nature of the intermolecular interactions, the thermodynamic properties of the gelation process, and the critical concentration of the precursors and the solvent will be reviewed. Because of their low toxicity, and potential applications in foods, cosmetics, drug delivery, tissue engineering and catalysis, attention will be directed to polysaccharide-based hydrogels with a special emphasis on CD-based host–guest hydrogel systems.

## 2. The Structure of Supramolecular Hydrogels

### 2.1. Design Strategy of Hydrogels: Mechanism of Gelation

Many gels are formed by simply heating a gelator or mixture of gelators in aqueous, organic or a co-solvent system to form a solution, followed by cooling. To gain a greater understanding of the mechanism of gel formation, the system may be categorized by the primary (1°), secondary (2°), and tertiary (3°) structure [64]. The 1° structure is determined by the molecular level recognition (e.g., host–guest interactions) that is largely influenced by nonspecific hydrophobic interactions. The 2° and 3° structures are determined by the molecular associations of individual polymer chains and their subsequent aggregation to form gels, respectively. In the case of host–guest systems, the formation of poly-pseudorotaxanes (PPRs) by threading a polymer chain into a series of CD cavities is one of the most common known self-assembly motifs in supramolecular hydrogels [54,65]. Strong hydrogen bonds between the adjacent PPRs function as non-covalent (physical) cross-links that lead to microcrystalline aggregation, thus promoting physical gel formation. A challenge for polysaccharide biopolymer gels concerns the molecular mechanism of non-covalent cross-linking in stimuli-reversible gelation. Generally, this is due to the role of non-covalent interactions; namely, H-bonding, hydrophobic and electrostatic interactions that result as the 3D networks are formed [66,67]. Moreover, the gelation process of many biopolymers is preceded by a transition from a random coil state to an ordered helix conformation, where the subsequent aggregation of the helices forms an extended network [67,68], as shown in the relationship below.

*Coil* → *Helix* → *Gel* (1)

**Scheme 1:**
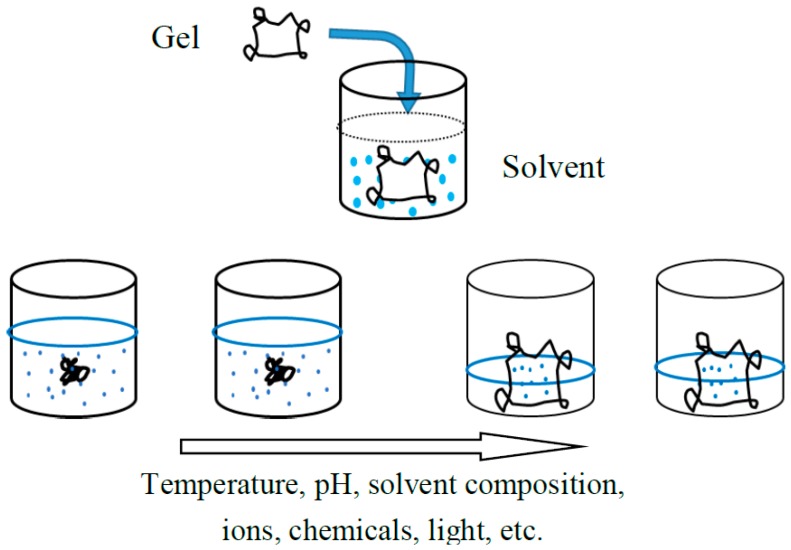
Phase transition of a gel indicated as a reversible and discontinuous volume change in response to various stimuli. Redrawn from [67].

The *coil–helix* (or *coil–globule*) transition in relationship (1) above is manifested as a volume phase transition (VPT) on a macroscopic level, where polymer gels can exist in either swollen or collapsed phases [35], as illustrated in Scheme 1.

**Figure 1:**
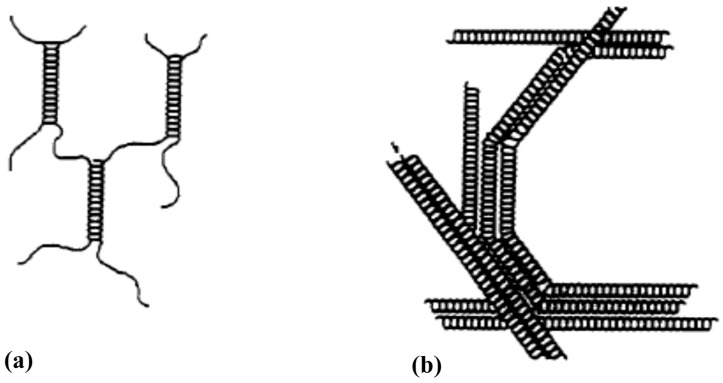
Schematic representations of possible mechanisms of network formation in helical gels: gelation on: (**a**) the helical level; and (**b**) super-helical level. Reproduced from [68] with permission. Copyright 1994 American Chemical Society.

VPT occurs between the phases in response to chemical and/or physical stimuli, such as temperature and pH, as a result of modification of the HLB of the system. For gelation to occur, the helix formation must lead to association and branching (or aggregation) of the polymer strands to form an infinite 3D network. Two possible mechanisms for association and branching occur as described in Figure 1. In Figure 1a, the association and branching may occur on the helical level, i.e., the formation of the helices and the aggregation of the polymer strands occur simultaneously. This is the classical mechanism of gelation which has been observed in many polysaccharide gels [47,68,69]. By comparison, Figure 1b represents a network formation at the super-helical level, where fully developed helices undergo self-assembly and aggregation to form a gel. Other more complex mechanisms which are beyond the scope of this mini-review have been proposed [70].

### 2.2. Volume Phase Transition (VPT) in Polymer Gels

Knowledge of the phase transitions in polymer gels is essential for establishing a greater understanding of the fundamentals underlying the associative interactions and molecular recognition in polymer networks. This effect occurs because the process of cross-linking of supramolecular assemblies in aqueous solution is known to result in VPTs as a function of external stimuli (cf. Scheme 1). The gel VPT was first predicted in 1968 by Dušek and Petterson, using the Flory–Huggins (FH) theory for polymer solutions [71]. A discontinuous volume change of a gel based on the analogy of the *coil–globule* transition of a polymer in a solution was reported, as depicted in Scheme 1. The thermodynamics of VPT of gels were described by Shibayama and Tanaka [72], where different competing interactions (e.g., Coulomb interactions between charged groups and counterions, van der Waals, hydrophobic, electrostatic, and hydrogen bonding interactions) control the gel size. For example, in the case of the volume phase transition of *N*-isopropylacrylamide (NIPA) gel [73], the enthalpic contribution due to hydrophobic interactions lead to gel collapse and entropic contributions due to the rubber elasticity that favour swelling of the gel. Therefore, gel size is determined by a simple additivity of the enthalpy/entropy contributions to the overall Gibbs energy of the process. The phenomenon of a gel phase transition resembles the gas-liquid phase transition. Thus, a discontinuous phase transition can be observed depending on the degree of ionizable groups and stiffness of the polymer chains that constitute the 3D network [18]. Theoretically, the swelling of a gel is determinable by minimizing the Gibbs energy per polymer segment with respect to polymer density or alternatively by modulating the pressure. The FH theory is an oversimplified mean-field theory which quantitatively describes the phase transition of a polymer network, where the Gibbs energy of a gel can generally be written as:

ΔG = ΔG_rubber_ + ΔG_counterion_ + ΔG_mixing_ (2)

**Scheme 2:**
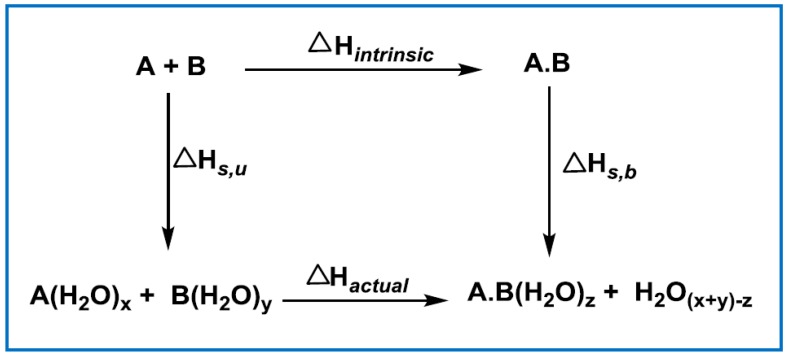
Born–Haber cycle showing separation of the actual (measured) enthalpy (ΔH_actual_) into intrinsic enthalpy (ΔH_intinsic_) and the enthalpy of solution values for the bound (ΔH_s,b_) and unbound species (ΔH_s,u_). The physical states of the reactants and products are not shown.

The terms on the right side of Equation (2) correspond to the Gibbs energy of rubber elasticity, ionization, and mixing, respectively. The terms for the total number of persistent (monomer) units and the polymer–solvent interaction energy, the number of ionized groups per chain, and a set of virial terms including the charge-charge repulsion, are contained within the generalized FH equation. Using the FH equation of state, Dušek and Patterson [71] concluded that a discontinuous volume change of a gel occurs when an external stress is imposed upon it. Generally, the VPT described by the oversimplified FH equation relate to polymer–solvent interactions and the tendency of the polymer chains to either repel (swell) or attract (contract) each other. Therefore, the role of the solvent in gel formation cannot be over emphasized. However, many studies have traditionally focused on solute–solute (intrinsic) interactions as the source of both binding enthalpy and recognition in the polymer self-assembly. In part, this relates to a limited understanding of the role of solvent effects in gel systems. The separation of the actual enthalpy of binding into an intrinsic (solute–solute) enthalpy (∆H_i_) and the enthalpies of solution for the bound (∆H_b_) and unbound (∆H_u_) species is shown by the Born–Haber cycle as presented in Scheme 2. The association of polymer chains in solution is therefore composed of solute- and solvent-associated processes.

### 2.3. Classification of Polysaccharide-Based Gels

Polysaccharide-based physical hydrogels can be classified according to various criteria [8,74,75,76], such as the source of the precursors, polymer composition, polymer configuration, type of cross-linking, and so on. In this mini-review, attention is made on the polymer composition as a basis to classify polysaccharide hydrogels. The method of gel preparation leads to the formation of some important classes of hydrogels, namely, homo-polymer, co-polymer, and multi-polymer inter-penetrating networks (IPNs).

#### 2.3.1. Single Component Homo-Polymer Gels

These types of gels are derived from a single species of monomer unit. Meena et al. [45] and Yoshida and Takahashi [49] reported χ-carrageenan and gellan as respective examples of polysaccharide-based single component thermo-reversible gels. Hydrogels reported by Jung et al. [51] represent single component polymer-based gels. Swellable functionalized starch and cellulose materials can also exist as 3D networks in water and may form hydrogels that are responsive to changes in temperature and pH [12]. Self-inclusion complexes of functionalized β-CD may exist as supramolecular polymers in water and undergo hydrogel formation [77]. The mechanism of the gelation in single component thermo-responsive hydrogels has been studied by X-ray diffraction and optical rotation studies. The process is based on the dissolution of the gelator material at high temperature, formation of double helices upon cooling, followed by aggregation of these helices [47], as described above. Stabilization of the double-helix is achieved by inter-chain H-bonding interactions. Gels based on aggregation of triple helices are also known [22]. Other examples of single component polymer gels were reported by Jejurikar et al. [42]. These gels were prepared from Ca^2+^ and Ba^2+^ cross-linked alginate materials. In the case of ionic cross-linked polymer gels, the structure that invokes the double helix is mimicked by cross-linking of specific polymers using multivalent counterion species such as Ca^2+^. Thermal induced formation of single component polymer-based gels were reported using chitosan derivatives [46] where such systems form due to the presence of hydrophobic interactions. The ability of such systems to form gels depends on the density and length of the hydrophobic side chains. Phase separation is related to hydrophobic chain segregation as the main mechanism of gel formation [46].

#### 2.3.2. Two Component Co-Polymer Gels

Co-polymer gels are composed of two types of interacting systems where one type is hydrophilic in nature. Host–guest complexes containing CDs (e.g., β-CD with cholesterol [78]) represent the simplest examples of co-polymer hydrogels formed from two polymers/components (where one or both are saccharides). Two component polymer gels can be formed from: (1) two non-gelling; (2) one gelling and one non-gelling; or (3) two gelling polymers. The mechanism of gel formation in this class of materials involves specific interactions (mostly H-bonding and/or hydrophobic, electrostatic, and π–π interactions) that give rise to gels whose properties depend on the structure of each polymer, as well as the relative concentration of each component [21]. In general, the structural characterization of the resulting gel due to the combination of two polysaccharides is dependent on variable factors. However, the structure will be governed partly by the kinetics of phase separation and the viscosity of the materials. An example of category (1) gels is a mixture of xanthan and galacto- or glucomannans. Examples of category (2) gels are dextran (non-gelling)/amylose (gelling) and galactomannan (non-gelling)/χ-carrageenan (gelling) mixtures [79]. The characterization of category (2) gels has been the subject of controversy among researchers. Some reports suggest that interpenetrating networks (IPNs; Section 2.3.3) of the two polymers are formed, while others suggest a phase separated entrapment of the non-gelling polymer within the gel network. Category (3) gels, e.g., agarose/χ-carrageenan mixture, form gels based mainly on IPNs. Evidence of IPNs relate to their formation by independent gelation of each polysaccharide, where a network of one polymer is formed followed by that of a second polymer in an independent fashion.

**Scheme 3:**
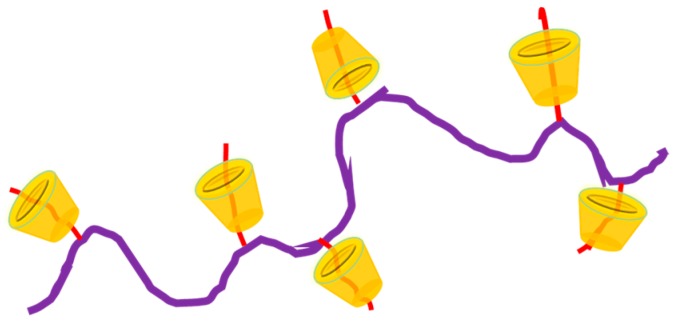
Hypothetical structure of a polymer network showing a chitosan backbone (purple line) with hydrophilic PEG pendants (red lines) and CD pendants (toroid).

Pseudo-copolymer systems based on multi-component host–guest polysaccharide-based gels have been extensively studied [1]. For example, Yui and coworkers [61,62] have prepared thermo-reversible supramolecular hydrogels using dextran or chitosan as hydrophilic backbones and short PEG or PPG side chains for inclusion complexation with CDs. The mechanism of gelation for these types of hydrogels is based on the phase separation of the hydrated backbone (e.g., dextrans or chitosan) and the hydrophobic-driven aggregation of inclusion complexes via physical cross-linking (cf. Scheme 3).

#### 2.3.3. Multi-Polymer Inter-Penetrating Networks (IPNs)

This category represents an important class of hydrogels combined of at least two polymers as a network assembly, where at least one component is independently synthesized and/or cross-linked in the presence of the other component without the formation of any covalent bonds [80]. Generally, IPNs are synthesized for the purpose of combining individual properties of two or more polymers. In many cases, new properties which are not observed in the single networks are observed in the prepared gel [43,81]. Various polysaccharides are used to prepare IPN-based gels, such as alginate, dextran, xanthan, chitosan, and guar gum [43,82].

### 2.4. Solvent–Gelator Interactions

Solvent interactions have a significant influence on the self-assembly of gels since the solvent constitutes about ~99% of the system by weight. Many biophysical processes in nature, such as enzyme–substrate interactions, the self-assembly of bilayers in biomembranes, surfactant aggregation, and kinetic solvent effects in water-rich solutions are predominantly governed by hydrophobic interactions [83]. Generally, the tendency of water molecules to avoid unfavourable entropic configurations with apolar solutes and constitutes a driving force for their aggregation. The self-assembly of gelators is a very important step in gel formation; however, the modulation of the HLB is equally important for ensuring that the 3D network imbibes the solvent within the polymer framework. Sparse studies [84] have reported the molecular level details of solvent effects in self-assembly and gelation processes. A variety of approaches by which the solvent effects can be quantified through physical parameters and equations have been reviewed in detail elsewhere [84,85]. A useful accounting method for specific solvent–solute interactions in gels involve the use of Kamlet–Taft parameters where α (hydrogen bond donor ability), β (hydrogen bond acceptor ability) and π* (polarizability) are defined [86]. Various studies have demonstrated that the α parameter strongly determines whether a hydrogen bonding gelator will undergo self-assembly in a given solvent [87,88]. Polar protic solvents with good hydrogen bond donating ability with high α values such as water, methanol or formic acid can interact competitively with the gelator. This leads to promotion of solute–solvent interactions and thwarts the formation of a gelator–gelator 3D network, leading to macroscopically homogeneous solutions. The β parameter represents the ability of the solvent to accept hydrogen bonds. Thus, solvents with high β values (e.g., tetrahydrofuran and ethyl acetate) can affect gelator–gelator interactions, resulting in a disruption of the self-assembly process, along with a lowering of the thermal stability of the gel network to a variable extent. In the case of the polarizability of a solvent (π*), the self-assembly and gelation can be modulated by the ability of the solvent to interact with the peripheral (surface or backbone) groups of the gelator, thus affecting the stability of the gel. Various structural factors that modulate the HLB need to be considered in order to modify solute–solvent interactions as a methodology for controlling gel formation (cf. Section 2.5).

### 2.5. Characterization of Hydrogels

Hydrogels and their stability are characterized by several methods including spectroscopy (e.g., nuclear magnetic resonance (NMR), Fourier transform infrared (FT-IR), ultra-violet visible (UV-vis), induced circular dichroism (ICD), and fluorescence), microscopy (e.g., scanning electron microscopy (SEM) and transmission electron microscopy (TEM)), diffraction (e.g., SAXS, SANS, and XRD), rheology (e.g., ball drop method and viscosity), calorimetry (e.g., DSC and ITC), and computational methods [89]. These techniques provide structural characterization of gels in terms of the nature of the intermolecular interactions, solute–solvent interactions, thermodynamics of the gelation process and the critical concentration of the gelators and the solvents. Selected examples of interest are briefly presented in this mini-review.

#### 2.5.1. Spectroscopy Techniques

Various techniques such NMR, UV-Vis, FT-IR, and Raman spectroscopy have been used to characterize the structure of hydrogels [15,24,29,54,84]. Solution- and solid-state NMR techniques at high resolution give useful structural information about the formation of H-bonds during gelation. The chemical shift changes of a specific nuclei during a *gel-sol* transition can be tracked using temperature dependent NMR measurements, where signals are broadened in the gel state [7]. Furthermore, chemical shift changes of a gel material can be used to probe local changes in a microenvironment such as aggregation [61,62]. ^13^C solid-state NMR can be used to study the 2° structure of gels giving an insight into their molecular arrangements [7]. UV-vis and FT-IR/Raman spectroscopy can be used to probe π–π interactions, and H-bonding, respectively. Although ICD may not directly support gel formation, it can be used as a supplementary technique to show evidence of double helix formation [90].

#### 2.5.2. Rheology

**Figure 2:**
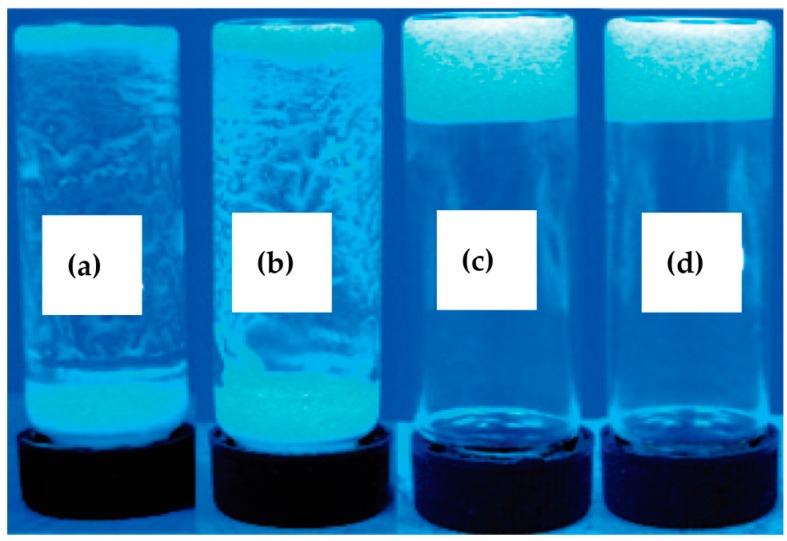
Optical photos of the complexes of: (**A**) mPEG1.1K; and (**B**) mPEG2K with α-CD; and invertible supramolecular hydrogels formed by: (**C**) Ada-PEG1.1K; and (**D**) Ada-PEG2K and α-CD. Reproduced from [65] with permission. Copyright 2008 American Chemical Society.

Rheology can be used to determine the mechanical properties of supramolecular hydrogels [90]. Gel structure has been characterized using such techniques as ball drop, inverted tube and modulus (viscosity) methods [91]. Despite its simplicity, the inverted-tube method has been used successfully to determine the stability of hydrogels. Figure 2 shows various solutions (PEG, PEG-α-CD, Ada-PEG, and Ada-PEG-α-CD) with different gel formation abilities as characterized by the inverted tube method. Note that the differences in structure of the mixtures shown in Figure 2 can be related to the HLB concept (cf. Scheme 3). Modulus methods have also been widely used to determine the strength of hydrogels [65,90], cross-over point of storage (G′) over loss moduli (G′′) and can be used to measure gelation kinetics of physical and chemical gel systems. Hence, by monitoring G′ and G′′, the viscoelasticity and the gelation time of hydrogels can be determined through variations of such factors as concentration and time. Stable gels are typically characterized by higher values of G′ over G′′.

#### 2.5.3. Diffraction Techniques

Diffraction techniques such as SAXS (small-angle X-ray scattering), SANS (small-angle neutron scattering) and XRD (X-ray diffraction) have been successfully applied in hydrogel characterization to elucidate their nanoscale structure and to provide insight on the molecular order of the system [92]. A typical example of hydrogel characterization by XRD was presented by Guo and co-workers (cf. Figure 9 in Reference [65]) for the adamantane (Ada)-polyethylene glycol (PEG)-α-CD hybrid supramolecular structure, where sharp XRD diffraction peaks ~2θ = 19.8° represent the extended channel structure of α-CD. The channel structure corresponds to the formation of α-CD-PEG inclusion complexes, which provides the driving force for the gel formation in this system, consistent with the mechanism of gelation in pseudo-copolymer multicomponent systems as depicted in Scheme 3 above.

#### 2.5.4. Microscopy Methods

Various microscopy methods such as Total Emission Microscopy (TEM), Scanning Electron Microscopy (SEM), Atomic Force Microscopy (AFM) and Scanning Tunnelling Microscopy (STM) have been used to characterize the morphology and microstructure of hydrogels [6,29,51,84]. TEM and SEM imaging can be used to visualize how small belts and fibres can entangle (aggregate) to form a 3D network. Thus, microscopy of such systems with variable morphology provide insight on the formation of H-bonds, π–π stacking, entangled networks, and other self-assembled structures [51]. While TEM and SEM give insight on the morphology of aggregation, AFM and STM are high resolution techniques that can be used to study the conformation of a gel [93]. Moreover, SEM/TEM [94] and AFM [95] can provide valuable information regarding the pore size and pore size distribution of such 3D networks.

#### 2.5.5. Modeling

**Figure 3:**
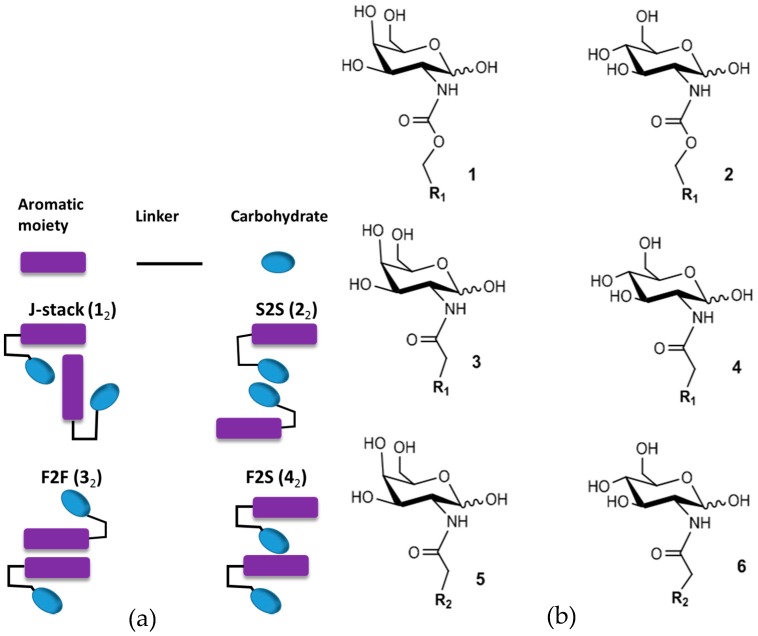
(**a**) Schematic representing dimer configurations of various amphiphilic systems (**1**–**6**); (**b**) Structures of the aromatic carbohydrate amphiphiles **1**–**6** containing different aromatic moieties (R_1_ and R_2_) and either a galactosamine or glucosamine residues. R_1_ and R_2_ represent fluorene and naphthalene residues, respectively. Redrawn from [90].

Computational techniques have been used to model the structural motif of supramolecular hydrogel networks [90,96]. Birchall and coworkers [90] successfully generated the structural features of some amphiphilic systems (**1–6**) that contain aromatic (fluorene or naphthalene) moieties and sugar (galactosamine or glucosamine) residues using molecular modeling [90] (cf. Figure 3b). Four possible modes by which dimers of four amphiphilic monomer units (**1**,**2**; **2**,**2**; **3**,**2**; and **4**,**2**) may be formed were proposed; (i) “J-stacking”, which involves a mixture of XH–π interactions between the aromatic residues (where X denotes a heteroatom); (ii) aromatic-aromatic π–π stacking (F2F); (iii) H-bonding between the sugar moieties (S2S); and (iv) solely XH–π interactions between the aromatic group and the sugar (F2S) (cf. Figure 3a). Each of the four possible configurations were optimized for the lowest energy structures and the most stable dimers were estimated from the relative binding energy of each pair. Molecular modeling can be used to determine if two or more systems form hydrogels based on whether the structural models display favourable binding energy value and their topology may lead to aggregation. The most favourable configuration for gel formation should minimize competitive solvent interactions and promote aggregation of the individual units whilst allowing the solvent to be imbibed within the 3D network. The terminal position of the sugar moieties in the F2F configuration (cf. Figure 3a) may promote competitive H-bonding with the solvent which limits the ability of the units to aggregate into a gel structure.

### 2.6. Improving the Stability and Performance of Hydrogels

In the foregoing sections, insight concerning the gelation process was revealed to gain a better understanding of how stimuli-responsive supramolecular hydrogels behave. One of the challenges of the design of supramolecular hydrogels is to modulate their physicochemical properties for optimum and specific applications. In the case of physical hydrogels where CD-based (host–guest) interactions are involved, the stability of the 3D network is variable and may significantly be weakened which limits the widespread application in areas of biomedical and environmental science. The destabilized structure of CD-based hydrogels is known owing to the weak non-covalent host–guest interactions along with the unfavourable entropy of binding of the restricted host and guest molecules within the polymer network. Selected physicochemical properties such as the stability of the 3D scaffold, the critical aggregation point, and the system viscosity can be manipulated to enhance the stability of hydrogels. Structural variations of this type have been achieved through design strategies that vary the nature/combination and feed ratios of the precursor materials, the use of amphiphiles or polymer inclusion complexes (PICs), and incorporation of nanoparticles (NPs), *vide infra*.

#### 2.6.1. Use of Polymer Inclusion Complexes (PIC)

The strategy of polymer inclusion complexation (PIC) is known to enhance the stability of supramolecular hydrogels. PIC-based supramolecular hydrogels involving polymers that contain cyclodextrin (CD) as an ideal host due to its hydrophobic inclusion sites have been widely investigated [48,53,57,61,62,63]. Small molecules or linear polymers can serve as guest molecules for the CD hosts. For example, PEG has been widely used since its first report in 1990 [96] to form supramolecular linear necklace-like PPR nanostructures with CD. Yui et al. [61,62] obtained a relatively low sol–gel transition temperature by using PIC formation between short PEG chains-modified chitosan (or dextran) and α-CD were synthesized by a series of coupling reactions. The phase-separation of the crystalline domains formed by the host–guest interaction between the α-CD and PEG provide the basis for understanding the supramolecular association and dissociation, namely; a sol–gel transition. The gelation properties of PIC-based hydrogels can be further tuned by adjusting the PEG content due to its variable hydrophilicity profile. Similarly, the solution concentration and the mixing ratio of the host and guest system may be varied. The optimal gel formation is a function of the fraction of crystalline PIC micro-domains, where an ideal host–guest stoichiometry will give the most stable network. At non-stoichiometric ratios, little or no gelation will be observed because of limited physical cross-linker domains. Hence, a delicate compromise between the host–guest stoichiometry and a chemical balance between components with variable hydrophile–lipophile balance (HLB) of the PIC system must be met for optimum gelation to occur.

#### 2.6.2. Use of Host–Guest Macromers (HGMs)

**Figure 4:**
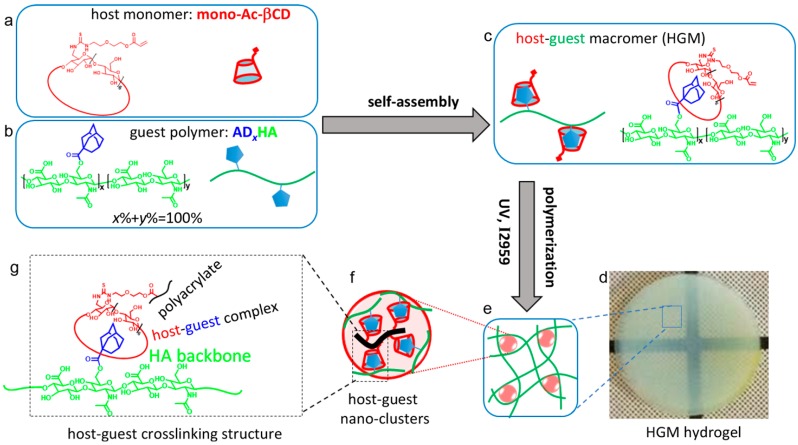
Chemical illustration of: (**a**) the host monomer (mono-Ac-βCD); (**b**) the guest polymer (AD_x_HA); and (**c**) the host–guest macromer (HGM). (**d**–**g**) Representation of various hydrogel/host–guest structures. Reproduced from [48] with permission. Copyright 2016 American Chemical Society.

The use of host–guest macromers (HGMs) [48] differ from PICs since the latter forms PPRs, whereas; macromers (cf. Figure 4c) are formed by HGMs. Basically, the HGM approach involves the self-assembly of pre-functionalized host and guest molecules. A previous report [48] on a responsive hydrogel formed from hyaluronic acid biopolymer functionalized with adamantane (AD) to form the guest polymer (AD_x_HA) and a polymerizable acrylate, (Ac)-functionalized β-CD (Ac-β-CD) as the host (cf. Figure 4). HGMs were reported to form via self-assembly between AD_x_HA and Ac-β-CD driven by efficient host–guest interactions of the less bulky host system and the guest polymer. The stability of the HGM-based hydrogels is understood due to the interaction between the macrocyclic host and guest within the HGM system, and is more favourable than those of the bulky polymer units. The resulting hydrogel [48] was found to be robust with potential utility as a carrier device with extended release properties.

#### 2.6.3. Use of Amphiphiles

The use of amphiphiles was long proposed [51,65,90] to promote the gelation process of hydrogels, along with an enhancement of gel stability through the HLB phenomenon, as described above. Co-polymers bearing Ada pendants as those described by Wei et al. [48] and Koopmans et al. [57] have been widely used to provide the hydrophobic end group requirement. A typical example of such association phenomena is the study reported by Guo et al. [65] where an aqueous solution of α-CD and LMW PEG undergoes precipitation over hydrogel formation (cf. Figure 3). This occurs when complexation occurs between α-CD and LMW PEG at both ends of the PEG, where the unbound PEG becomes too short to form a network. However, when the PEG was functionalized with the highly hydrophobic Ada, a gel was formed. The Ada group serves to (i) decrease the amount of threaded CD which provides sufficient unbound PEG; and (ii) provide additional physical cross-links via hydrophobic aggregation. On this basis, it can be concluded that for hydrogels which are composed of an amphiphilic block copolymer and a CD, the driving force for gelation is a combination of inclusion complexation between CD and PEG blocks, as well as the aggregation of the hydrophobic Ada blocks via favourable interactions illustrated in Scheme 3. Jung et al. [51] reported the first example of a hydrogel formation using a sugar-based amphiphile, where well-defined bilayer aggregates self-assemble via intermolecular hydrogen bonding, π–π stacking and hydrophobic interactions. The synergetic role of various interactions is essential for the successful design and stabilization of such hydrogel systems.

#### 2.6.4. Use of Hybrid Hydrogels and Nano-Fillers

The incorporation of nanoparticles (NPs) has emerged as one of the latest strategies of modulating the mechanical strength and the viscosity of supramolecular hydrogels. Guo et al. [65] used modified β-CD-silica NPs (β-CD-SiO_2_) to enhance the gelation of low molecular weight (LMW) PEG-α-CD hydrogels. Cooperative binding of the hydrogel and the NPs yield a strong network structure that leads to a nanoparticle-hybridized supramolecular hydrogel. The storage modulus (G′) and the viscosity of the hybrid hydrogel containing ca. 9 wt % of the modified NPs were increased by ca. 4- and 10-fold relative to the native hydrogel. The effect of the incorporation of silver and ferrous NPs into the supramolecular hydrogel networks was reported to enhance their stability. Ma et al. [92,97] reported a PEG-PCL(poly-ε-caprolactone)-α-CD hydrogel hybridized with magnetic Fe_3_O_4_ NPs. The introduction of magnetic Fe_3_O_4_ NPs in PEG-PCL-α-CD hydrogel was concluded to speed up the gelation time and to improve the stability of the hydrogel nanocomposite. The interaction of PEG-PCL with the dispersed Fe_3_O_4_ provided favourable conditions for the subsequent complexation with α-CD. Wang et al. [98] reported hydrogel systems from PEO-PPO-PEO triblock copolymers, α-CD and an inorganic nanotube. The addition of the nanotube resulted in a suppressed hydrophobic aggregation of the middle PPO block lowering the viscosity of the final hydrogel. Thus, the introduction of NPs can be used as a way to modulate the stability of hydrogels for tailored applications.

### 2.7. Modulating the Viscosity: Influence on Host–Guest Complexation

Various studies have indicated that hydrogel stability generally increase as the gelator concentration increases [57,60,96]. This effect indicates that the formation of hydrogels with stronger networks occurs as a result of more extensive H-bonding and efficient π–π stacking of the hydrogelators. Supramolecular hydrogels investigated by Koopmans and Ritter [57] represent typical examples that reveal the effects of viscosity, concentration and pH in modulating hydrogel stability. Their report studied a series of hydrogel systems based on acrylamide-Ada polymers bearing variable spacer units, as host molecules, and epichlorohydrin-CD globular/linear copolymers as host molecules, respectively (cf. Scheme 3 in Reference [57]). The effects of host/guest polymer concentration, length/amount of hydrophobic Ada chains/groups, and the amount of cross-linker for the α-CD on the stability of the hydrogel were investigated. pH effects were reported to alter the viscosity of the hydrogel. The study by Koopmans and Ritter [57] demonstrate that the viscosity of the medium can affect the stability of a hydrogel network, which reaches a maximum value at specific host–guest stoichiometry, temperature and pH conditions reported therein. Thus, the stability of a hydrogel can be tuned to specific applications by varying the concentration of the host/guest system. Apart from the host/guest concentration, the hydrogel viscosity and its stability depend on other factors that can influence host–guest complexation. For example, the HLB effect (e.g., the length/number of the hydrophobic Ada moiety/groups), amount of cross-linker used for the host molecule (in the case of cross-linked hosts), the pH of the hydrogel medium, and the type of host polymer (*globular vs. linear*).

## 3. Conclusions

In this mini-review, a general outline describing the mechanism of gel formation in polysaccharide-based supramolecular hydrogels was presented. While the stabilization of helices is the generally proposed pathway of gel formation in single polymer networks, phase separation of polymer networks is supported by the overall mechanism of *gel* → *sol* transition in polymer networks. The Flory–Huggins relationship and the utility of the Born–Haber cycle provide insight on the molecular level cross-linking of polymers in aqueous solution. By comparison, the gel formation process involves solute- and solvent-associated steps. As well, several strategies were outlined that can be used to fine-tune the physicochemical properties of biopolymer networks. Many of these strategies relate to stabilizing the 3D structure of the polymer scaffold in such gels as follows: (1) controlling the HLB of the polymer system; (2) controlling the cross-linking of the co-polymers; (3) providing favourable conditions for host–guest complexation; and (4) controlling independent variables such as pH of the medium, along with the nature and concentration of the precursors. Generally, the process of gelation in biopolymers is poorly understood due to inadequate understanding of the role of solvation phenomena (thermodynamic, kinetic, and structural effects) in aqueous media. The role of solvation phenomena in gel formation processes is a suggested direction of future research that deserves further attention.

## Figures and Tables

**Table 1:** Selected examples of polysaccharide biopolymers reported for the preparation of hydrogels.

Origin	Gelator/Precursor	Responsive Feature	References
Plant cell walls, wood, seeds, & roots	Pectins, cellulose, galacto-/gluco-mannans	Chemical species (arsenic), pH, & temperature	[21,25,26,41]
Seaweeds	Carrageenans, alginates, agar	Light, & temperature	[5,42,43,44,45]
Animals, organisms, bacteria	Hyaluronan, chitosan, chondroitins, xanthan, succinoglycan, gelatin, gellan	Temperature, & pH	[19,46,47,48,49,50]
Sugars	Cyclodextrins, galactose, glucose	Redox, light, temperature, & chemical species	[25,51,52,53,54]

## Abbreviations

3D	Three dimensional
Ada or AD	Adamantane
CD	Cyclodextrin
DSC	Differential scanning calorimetry
FT-IR	Fourier transform infra-red
HGM	Host–guest macromer
HLB	Hydrophile lipophile balance
ICD	Induced circular dichroism
IPN	Interpenetrating network
LMW	Low molecular weight
NMR	Nuclear magnetic resonance
NP	Nanoparticle
PEG	Polyethylene glycol
PIC	Polymer inclusion network
PPR	Polypseudorotaxane
SANS	Small-angle neutron scattering
SAXS	Small-angle X-ray scattering
SEM	Scanning electron microscopy
TEM	Total emission microscopy
STM	Scanning Tunneling Microscopy
UV/Vis	Ultra-violet visible
XRD	X-ray diffraction
